# Genomic Survey, Transcriptome, and Metabolome Analysis of *Apocynum venetum* and *Apocynum hendersonii* to Reveal Major Flavonoid Biosynthesis Pathways

**DOI:** 10.3390/metabo9120296

**Published:** 2019-12-05

**Authors:** Gang Gao, Ping Chen, Jikang Chen, Kunmei Chen, Xiaofei Wang, Aminu Shehu Abubakar, Ning Liu, Chunming Yu, Aiguo Zhu

**Affiliations:** Institute of Bast Fiber Crops, Chinese Academy of Agricultural Sciences, Changsha 410205, China; gaogang@caas.cn (G.G.); Chenping02@caas.cn (P.C.); chenjikang@caas.cn (J.C.); Chenkunmei@caas.cn (K.C.); xiaofei1008@126.com (X.W.); aashehu.agr@buk.edu.ng (A.S.A.); liu7ning2628@163.com (N.L.)

**Keywords:** *Apocynum venetum*, *Apocynum hendersonii*, genomic survey, flavonoids, metabolites profiles, gene expression

## Abstract

*Apocynum* plants, especially *A. venetum* and *A. hendersonii,* are rich in flavonoids. In the present study, a whole genome survey of the two species was initially carried out to optimize the flavonoid biosynthesis-correlated gene mining. Then, the metabolome and transcriptome analyses were combined to elucidate the flavonoid biosynthesis pathways. Both species have small genome sizes of 232.80 Mb (*A. venetum*) and 233.74 Mb (*A. hendersonii*) and showed similar metabolite profiles with flavonols being the main differentiated flavonoids between the two specie. Positive correlation of gene expression levels (flavonone-3 hydroxylase, anthocyanidin reductase, and flavonoid 3-O-glucosyltransferase) and total flavonoid content were observed. The contents of quercitrin, hyperoside, and total anthocyanin in *A. venetum* were found to be much higher than in *A. hendersonii,* and such was thought to be the reason for the morphological difference in color of *A. venetum* and *A. hendersonii*. This study provides valuable genomic and metabolome information for understanding of *A. venetum* and *A. hendersonii*, and lays a foundation for elucidating *Apocynum* genus plant flavonoid biosynthesis.

## 1. Introduction

*Apocynum*, commonly known as kendir or Luobuma since pre-historic times due to its excellent quality fiber, is a genus in the Apocynaceae family used as a medicine and source of fiber. They are considered native to a large number of countries in northern and south Eastern Europe, northern America, and Asia. Although there is much divergence in their taxonomy, there exist mainly two species, *Apocynum venetum* and *Apocynum hendersonii* (also called *Apocynum pictum*) in China distributed widely in the northwest and the coast of the Yellow Sea [1,2]. Both species are eco-economic perennial rhizomatous plants with high stress resistance and economic value, with rhizomes extending up to 5–6 m horizontally underground, and adapted to survive in extreme environments of low rainfall, saline and alkaline deserts, river banks, alluvial plains, and the Gobi desert [3].

*A. venetum,* in addition to serving as source of bast fiber, is very well known for possessing broad medicinal uses as anti-platelets [4], anti-depressive [5], anti-hypertensive [6], anti-oxidant [7], anti-aging [8], anxiolytic activity [1], stimulant as tea [9] etc. Meanwhile, *A. hindersonii* is morphologically similar to *A. venetum* but with a major visible distinction being in the color of stem and leaf, and the shape of their flower, owing likely to their local names “White Hemp” and “Red Hemp”, respectively.

Applications of *A. hendersonii* are usually limited to fibers for spinning and paper making, rather than medicinal purpose [8], due to the relative low total flavonoid content. Chan et al. tentatively identified ten kinds of flavonoid components by ultra-visible and mass spectra, and hyperoside was identified as a critical parameter or a suitable chemical marker for discrimination of two species [7]. However, as *A. venetum* and *A. hendersonii* are distributed widely from the northwest to the coast of the Yellow Sea in China, the tremendous differences of the natural conditions may lead to the great differences in phenotype, structure, and chemical compositions. The whole biological effects of both plants are borne out by their bast fiber and flavonoids [10].

Recently, researches on the two species have attracted widespread attention. Previous researches were mainly focused on the identification of morphological characteristics or extraction of phytochemicals, and combinative technologies of bulked segregate analysis (BSA) coupled with randomly amplified polymorphic DNA (RAPD) were developed to distinguish the two species [2]. Yet very few studies have assessed the genetics of *A. venetum* and *A. hendersonii,* and inadequacy of gene sequence resources have limited their in-depth researches. Flavonoids play important roles in a wide range of physiological processes. In China and Japan, the leaves of *A. Venetum* and *A. hendersonii* are used as traditional herbal teas due to their higher flavonoid content than *Camellia sinensis* [9]. Flavonoids are synthesized through a long pathway, which has largely been characterized in *Arabidopsis thaliana* and *Zea mays*. Briefly, the upstream pathway consists in the formation of the core, the basic skeleton of all flavonoids (starting from malonyl-CoA and 4-coumaroyl-CoA to naringenin). The oxidation of naringenin yields the dihydrokaempferol that can subsequently be hydroxylated, producing, respectively, dihydroquercetin or dihydromyricetin [11,12,13]. Researchers have tended to focus on specific compounds, rather than thoroughly assessing all of the constituents. Previous studies found that the content of flavonoid in *A. hendersonii* was lower than in *A. Venetum*. So, identifying and investigating the regulatory mechanisms behind flavonoid biosynthesis is essential to understanding their roles in *Apocynum* to improve flavonoid production by metabolic engineering, and a comparative analysis of their chemical composition or pharmacological activities is certainly needed.

In order to analyze crucial genes in the synthesis of flavonoids and fully mine molecular biology data from *A. venetum* and *A. hendersonii*, we present here the metabolome profile and transcriptome data to analyze metabolite pathways associated with flavonoid biosynthesis. In addition, we conducted a genomic survey of *A. venetum* and *A. hendersonii*. The availability of genomic information may provide an opportunity to elucidate genes involved in the biosynthesis of flavonoids, and also have significance for genomics and evolutionary research of *Apocynum* genus plants.

## 2. Results and Discussion

### 2.1. Genome Survey of A. venetum and A. hendersonii

A total of 116.45 Gb for *A. venetum* and 120.61 Gb for *A. hendersonii* raw base were obtained by the Illumina Hiseq platform (Table 1). After filtering low-quality data, the clean read rates of 98.12% and 97.34% raw base were obtained for *A. venetum* and *A. hindersonii,* respectively. The sequencing quality evaluation result also shows raw Q30 base rate (92.16%) and clean Q30 base rate (92.75%) for *A. venetum*. Similarly, raw Q30 base rate (91.92%) and clean Q30 base rate (92.21%) were recorded for *A. hendersonii,* indicating high quality and accuracy of the high-throughput sequencing.

The clean bases were used for preliminary genome assembly, and K-mer value of 35 was selected for constructing contig and scaffold (Table 2). In *A. venetum*, we obtained a total of 1250,389 contigs with a total length of 322,394,863 bp and the longest sequence assembled of 6632 bp. The length of the N50 Contig was 310 bp. The number of obtained scaffolds was 876,453, having a total length of 377,590,884 bp, and thr longest sequence assembled of 70,393 bp and N50 scaffold of 1225 bp. A total of 741,647 contigs with a total length of 199,859,654 bp was obtained for *A. hendersonii*, with the longest sequence assembled of 8521 bp and 457-bp-long N50 Contig. A total of 303,264 scaffolds were obtained after further assembly with a total length of 236,656,356 bp, and the longest sequence assembled of 110,436 bp. The length of the N50 scaffold was 4667 bp.

Following exclusion of error effects due to erroneous K-mers (Table 3), the genome sizes were found to be 232.80 Mbp and 233.74 Mbp for *A. venetum* and *A. hendersonii,* respectively. *A. venetum* and *A. hendersonii* genome sizes are, therefore, relatively small in comparison to published genome sizes of other plants [14,15], suggesting future genome assembly and comparative studies on genome evolution to also be relatively simple. Heterozygosity is an important feature of plant genome, and many crop species are manipulated to increase heterozygosity for heterosis breeding [16,17]. Calculating the proportion of heterozygous sites in the sequences of both species revealed a gene heterozygosity ratio of 0.67% and 0.46% for *A. venetum* and *A. hendersonii,* respectively, indicating more heterozygous complexity in *A. venetum*.

### 2.2. Transcriptome Sequencing, De Novo Assembly, and Pathway Analysis

RNA libraries were derived from *A. venetum* and *A. hendersonii* leaves and were sequenced using Illumina HiSeq2500. A total of 47.9 million clean reads with 44.2% GC content (guanine plus cytosine content) for *A. venetum* were acquired, and FastQC analysis show 95.3% of the total sequences to possess a quality above Q30. RNA-seq libraries for *A. hendersonii* yielded 48.1 million clean reads with 44.1% GC content and FastQC analysis show 95.4% of the total sequences with quality above Q30.

**Figure 1:**
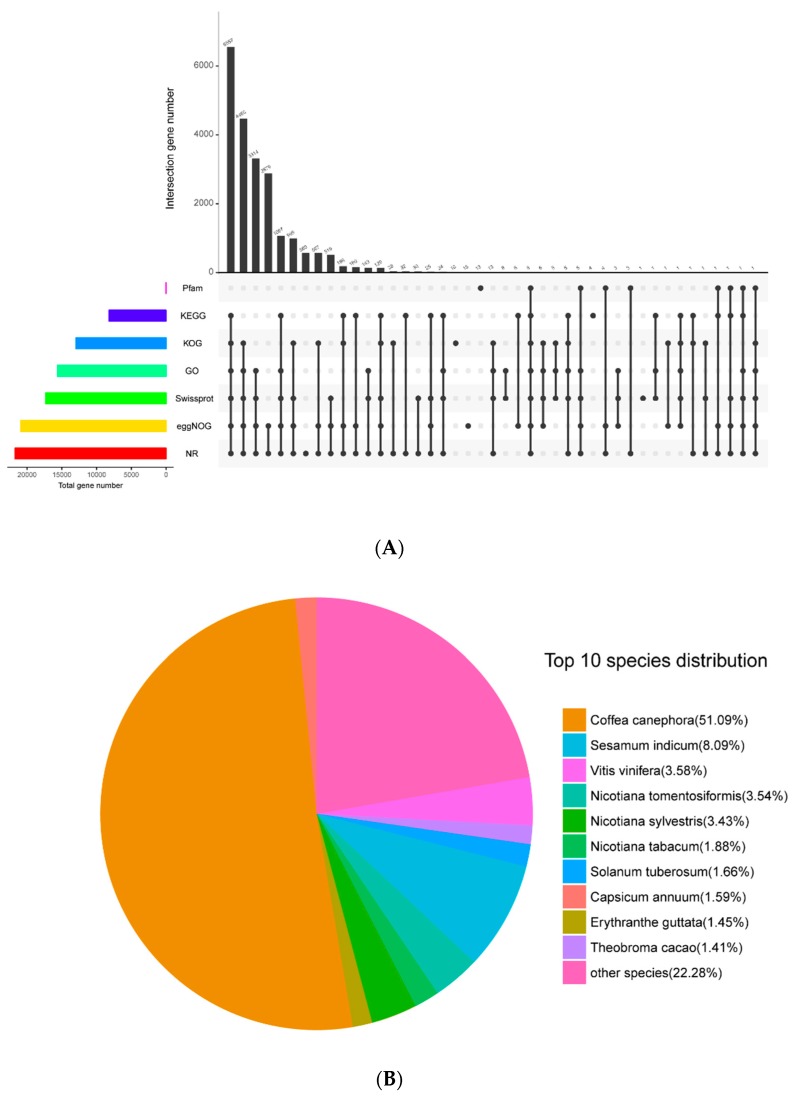
Venngraph (**A**) of *A. venetum* and *A. hendersonii* unigenes annotated against seven databases and the species distribution on the top BLASTX hits (**B**). (KOG, Clusters of orthologous groups for eukaryotic complete genomes; KEGG, Kyoto encyclopedia of genes and genomes; GO, Gene ontology; NR, Non-redundant protein sequence databas; eggNGO, Evolutionary genealogy of genes: Non-supervised orthologous groups).

Pooled reads of both species were assembled de novo into a total of 32,740 unigenes with N50 of 2081 bp. All of the unigenes were annotated by aligning with public databases and obtained 21,743 (66.41%) significant hits in Nr; 17,328 (52.93%) in Swiss-Prot; 8211 (25.08%) in KEGG (Kyoto encyclopedia of genes and genomes); 12,995 (36.69%) in KOG (Clusters of orthologous groups for eukaryotic complete genomes); 20,918 (63.89%) in eggnog; 15,627 (47.73%) in GO (Gene Ontology); and 37 (0.11%) in Pfam databases. The best-match result of species distribution (Figure 1) gave top hits of 51.09% of unigenes to be from *Coffea canephora,* followed by *Sesamum indicum* (8.09%), *Vitis vinifera* (3.58%), *Nicotiana tomentosiformis* (3.54%), and *Nicotiana sylvestris* (3.43%). The 15,627 unigenes reflected in GO were assigned to one or more terms and categorized into 60 functional groups under three main partitions, namely, biological process, molecular function, and cellular component. Of the unigenes, 10,461 (66.94%) in the biological process partition fall under highly encoded proteins that are involved in the cellular process, followed by 9064 unigenes (58.00%) involved in the metabolic process, 4670 unigenes (29.88%) in biological regulation, and 4566 unigenes (29.21%) involved in response to stimulus.

A total of 14538 unigenes were also searched against the KOG database and clustered into 26 classes. The largest category, 2990 unigenes (20.56%), fall into general function prediction, followed by post-translational modification, protein turnover, chaperones (1469 unigenes, 10.10%), and signaltransduction mechanisms (1236 unigenes, 8.50%). Moreover, 577 unigenes were classified as secondary metabolite biosynthesis, transport, and catabolism. These results opined that both *A. venetum* and *A. hendersonii* undergo extensive metabolic activity. Additionally, pathway analysis of differentially expressed genes (*A. venetum* vs. *A. hendersonii*) identified flavonoid biosynthesis (ko00941) and flavone and flavonol biosynthesis (ko00944) as the top 20 up-regulated KEEG pathways [18], suggesting higher flavonoid content in *A. venetum*.

### 2.3. Metabolic Profiles of A. venetum and A. hendersonii

**Figure 2:**
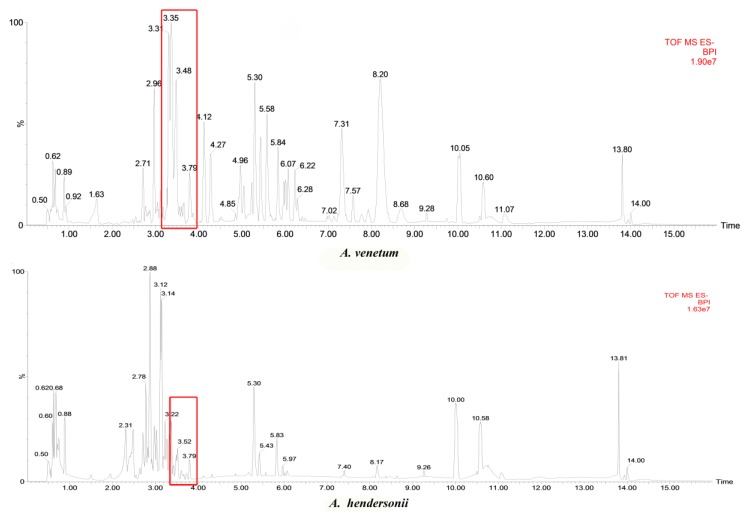
Typical base peak intensity (BPI) chromatograms showing metabolic profiles of *A. venetum* and *A. hendersonii* leaf in the negative ionization mode.

We performed a comparative metabolomics analysis between the two species based on the LC/MS (liquid chromatograph-mass spectrometer) data obtained from the individual species leaf. The results show that both of the *A. venetum* and *A. hendersonii* were rich in secondary metabolites, and more than 7500 kinds of components were detected and identified in the two species. Positive and negative ionization modes were used to evaluate the results, while the negative ionization mode being more selective and sensitive for presenting metabolomic profile with higher number of compounds and intensities [19] was chosen for further analysis. Upon comparison of observed *m/z* values with data of mass spectra of agrochemicals, pharmaceuticals, and physiologically active compounds (including drugs, steroid hormones, or endocrine disruptors) from mass spectral libraries NIST and WILEY (https://www.sisweb.com/software/ms/wiley.htm), several peaks were detected. In this study, the main peaks identified as flavonoids are indicated in Figure 2 using the red box (3.3139, delphinidin; 3.3143, quercetin 3-(6’’-malonyl-glucoside); 3.31393, quercetin 3-galactoside; 3.31393, kaempferol 3-(6-acetylgalactoside); 3.31435, isoorientin; 3.48303, Luteolin; 3.48795, kaempferol 3-O-arabinoside; 4.0666, kaempferol; 3.4302, rutin; 3.55095, tamarixetin). Other flavones or anthocyanins detected were: 11.85368, pelargonidin; 11.26885, malvidin; 10.82683, peonidin; and 10.37761, luteolin. When taking the contents of the flavonoid constituents into account, it was found that there indeed existed quite a few differences between the two species. As is known, flavonoids may promote desirable and non-desirable physiological effects in humans or animals, and their biological activity is largely determined by the concentrations. This comprehensive and unique phytochemical profile study revealed the diversity of secondary metabolites (especially flavonoids) between *A. venetum* and *A. hendersonii*; correlation studies between potential markers and biological activities assays need to be conducted in the future.

**Figure 3:**
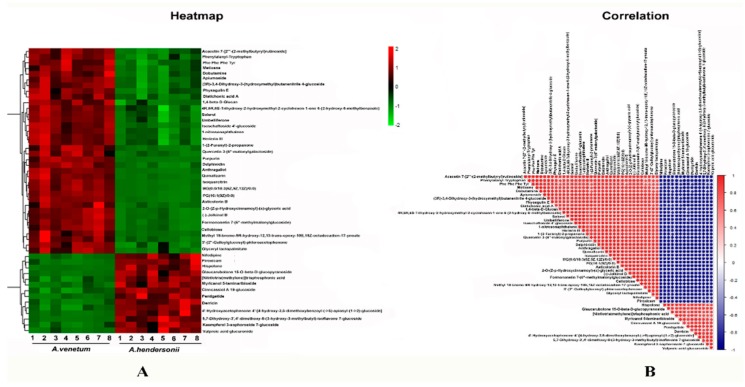
Heatmap (**A**) and correlation (**B**) among the top 50 differentially accumulated metabolites between *A. venetum* and *A. hendersonii*. The content of each metabolite was normalized to complete linkage hierarchical clustering. Each example in (**A**) is visualized in a single column and each metabolite is represented by a single row. Red indicates high abundance, whereas low relative metabolites are shown in green. The red and blue color in (**B**) indicate the positive and negative correlations, respectively.

The data obtained were initially submitted to a PCA (Principal Component Analysis) approach using the Pareto scale. The secondary metabolites were clearly separated and grouped into two clusters corresponding to *A. venetum* and *A. hindersonii*. The differential metabolites were selected on the basis of the combination of a statistically significant threshold of variable influence on projection (VIP) values obtained from the OPLS-DA (Orthogonal partial least squares discriminant analysis) model and *p*-values from a two-tailed Student’s *t*-test on the normalized peak areas (Figure S1). We observed that in both the *A. venetum* and *A. hendersonii* leaf, the general pattern of metabolites was relatively similar, while the accumulation content of 665 metabolites such as flavonoids and sterols was found to be significantly different. These compounds could be used to explore the potential chemical markers that contribute to the diversity between the two species. The hierarchical clustering and correlation analysis of the top 50 differentially accumulated metabolites between *A. venetum* and *A. hendersonii* are shown in Figure 3, which visually shows the intensities of the potential chemical biomarkers between *A. venetum* and *A. hendersonii*.

### 2.4. Major Flavonoids Identified in A. venetum and A. hendersonii

Flavonoids, which are widespread in plants and fruits, have lots of structural variations that can be subdivided into six major groups according to the hydroxylation pattern and conjugation between the aromatic rings; namely, flavones, flavanones, flavonols, flavanols, anthocyanins, and isoflavones. In our analyzed leaf extracts, two flavones (luteolin, apigenin), six flavonols (rutin, hyperin, isoquercitrin, quercetin, kaempferol, tamarixetin), three flavanols (catechin, epicatechin, epigallocatechin), one flavanones (hesperidin), one isoflavanone (trifolin), seven anthocyanidins (cyanidin, procyanidin c1, procyanidin, delphinidin, pelargonidin, malvidin, peonidin), and two chalcones (carthamin, neocarthamin) were found in *A. venetum* and *A. hendersonii* leaves. A few other flavonoids (acacetin, chrysoeriol 7-O-glucoside, acacetin-7-O-rutinoside) not detected before were also identified. The chemical characterizations of the flavonoids are described in Table 4. Among these compounds, flavonols are the major flavonoids. This result is consistent with the previous data [20,21], while more anthocyanins were detected.

Quantitative analysis shows that flavonols are the main differentiated flavonoids between the *A. venetum* and *A. hendersonii* leaf samples. The content of hyperin, isoquercitrin, quercetin, kaempferol, and rutin in *A. venetum* was much larger than that in *A. hendersonii*—evidence of its higher flavonol content. This result conforms to the reports that hyperoside and isoquercetrin could serve as good chemical markers to differentiate the two species [22].

### 2.5. Analysis of Flavonoids Biosynthesis-Related Transcript Level and Metabolites Content

The flavonoid pathway consists of a number of enzymatic steps, each catalyzing a sequential reaction. Phenylpropanoid is a direct precursor for the synthesis of anthocyanins and other flavonoids. The basic skeleton of all flavonoids, starting from three molecules of malonyl-CoA and one of 4-coumaroyl-CoA, produces a colorless flavanone named naringenin. The related enzymes, including phenylalanine ammonialyase (PAL, Gene ID: *DN7770*, *DN12095*), cinnamic acid 4-hydroxylase (C4H, Gene ID: *DN15816*), chalcone isomerase (CHI, Gene ID: *DN13226*, *DN3483*, and *DN8427*), and chalcone synthase (CHS, Gene ID: *DN18628*), were identified in both *A. venetum* and *A. hendersonii*. Transcriptome analysis revealed there was no significant difference in the expression level of these genes, and the content of metabolites such as naringenin that were detected in the first few biosynthesis pathway of both *A. venetum* and *A. hendersonii,* which formatted the core and basic skeleton of flavonoids, also did not show significant difference.

**Figure 4:**
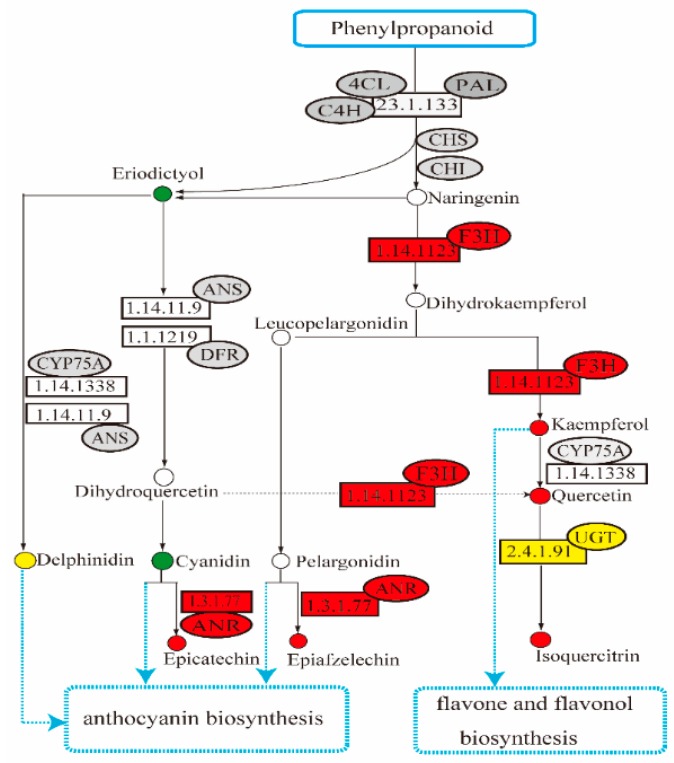
Simplified representation of the enrichment of different expressed flavonoid biosynthetic pathway genes of *A. venetum* vs. *A. hendersonii*. (4CL, Coumarate Coenzyme A Ligase; PAL, Phenylalanine ammonialyase; 4CH, Cinnamic acid 4-hydroxylase; CHI, Chalcone isomerase; CHS, Chalcone synthase; F3H, Flavonol 3-hydroxylase; ANR, Anthocyanidin reductase; DFR, Dihydroflavonol 4-reductase; CYP75A, Subfamily of cytocrome P450; UGT, Flavonoid 3-O-glucosyltransferase; ANS, Anthocyanidin synthase).

The subsequent reactions in the flavonoid biosynthesis pathway are from naringenin to dihydroflavonol, anthocyanins, flavonols, or other flavonoids with the different enzymes. Among these key enzymes, two flavonol 3-hydroxylase (F3H)-encoding genes (ID: *DN13255* and *DN9170*), three anthocyanidin reductase (ANR)-encoding genes (ID: *DN11675*, *DN11945*, and *DN20760*), five flavonoid 3-O-glucosyltransferase-encoding genes (ID: *DN11850*, *DN14334*, *DN16440*, *DN19250*, and *DN6934*) were identified, and their expression levels were significantly up-regulated in *A. venetum* rather than in *A. hendersonii*. Other differentially expressed genes, such as kaempferol 3-O-beta-D-galactosyltransferase (ID: *DN10026*) and leucoanthocyanidin reductase (ID: *DN11675*) encoding genes, were also identified with a log2 fold change value of 5.08 and 1.65 in *A. venetum* vs. *A. hendersonii* samples, respectively. The content of corresponding products (kacmpferol, quercetin, isoqucrcitrin, epicatechin, and epiafzelechin) in *A. venetum* were also higher than in *A. hendersonii*. Further confirmation by quantitative PCR (qPCR) analysis shows that all of the selected genes had the same expression pattern, revealed by the differential analysis results from high throughput sequencing (Table S1). The positive correlation between the transcript level and total flavonoid content (Figure 4) obtained in this study indicates that accumulation of flavonoids was accompanied by the induction of flavanone 3-hydroxylase, anthocyanidin reductase, and flavonol 3-O-glucosyltransferase.

**Figure 5:**
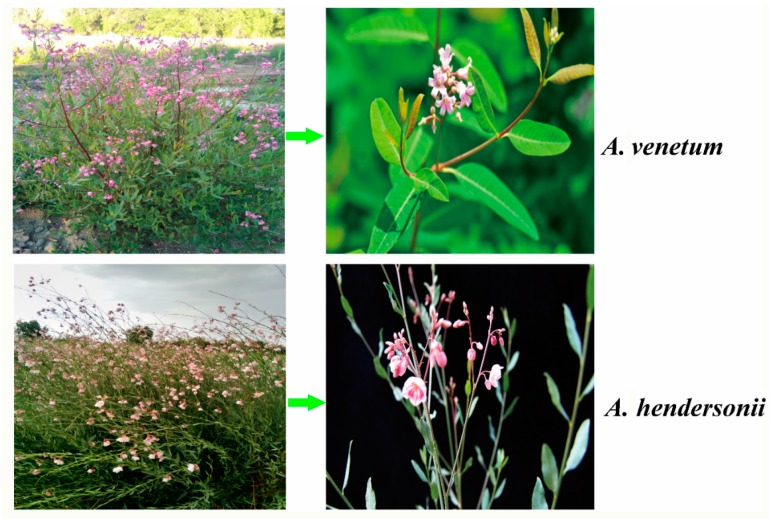
Phenotypes and morphology of *A. venetum* and *A. hendersonii* leaf, stem, and flower. The stem and leaf of *A. venetum* show more of a dark red phenotype than *A. hendersonii*.

The flavonoid biosynthesis pathway is one of the important pathways influencing *Apocynum* quality, with the biosynthesis starting from phenylalanine, then channeled to different branches of the pathway to yield many different compounds. Anthocyanins, one of the products of the pathway, are a class of water-soluble pigments providing certain color for various plant parts [13,22,23]. Transcription factors regulating anthocyanin synthesis via different mechanisms were previously identified in plants with dark- or light-colored phenotype of fruit or other organs, such as red-skinned and red-fleshed apple, purple and green asparagus or eggplants. The coloring of red-skinned apples is primarily determined by anthocyanin [24,25,26,27,28]. Purple and green asparagus cultivars were analyzed for anthocyanins biosynthetic and regulatory genes, and the purple asparagus was reported to have much higher anthocyanin than all other purple vegetables [29]. Our transcriptome and metabolome content correlation analyses show higher transcriptomic and metabolomic levels related to anthocyanins in *A. venetum* than in *A. hendersonii*, especially the anthocyanidin reductase genes which play roles in the early unbranched segment of the flavonoid biosynthetic pathway were found to be significantly up-regulated in *A. venetum*. We conclude that the complexity of the regulation mechanism of flavonoid biosynthesis is the embodiment of the diversity between *A. venetum* and *A. hendersonii,* and the red coloring of *A. venetum* (Figure 5) may be primarily determined by anthocyanins.

The HPLC (High performance liquid chromatography) method was adopted for quantitative analysis of hypersoside, isoquercitrin, quercetin, and rutin. The results show the amount of flavonoids compounds selected in the two species to have varied tremendously in accordance with the LC/MC profile (Table 5). The highest level of isoquercitrin (5.254 mg/g of DW, dry weight) was obtained in *A. venetum,* which was significantly higher than that obtained in *A. hendersonii* (1.718 mg/g of DW), suggesting that, in addition to hyperoside [7], isoquercitrin could also be a good chemical marker to differentiate *A. venetum* and *A. hendersonii*. The amount of total anthocyanins was determined using the previously described pH differential method. The average content of total anthocyanin in *A. venetum* was also much higher than in *A. hendersonii*. The amount of anthocyanin in *A. venetum* leaves (0.9017 mg/g) was comparable to the amount reported in *Rosa rugose* (1.1790 mg/g), *Rosa chinensis* (1.1010 mg/g), *Hibiscus sabdariffa* (0.8610 mg/g), *Camellia japonica* (0.8280 mg/g), and *Dianthus caryophyllus* (0.8180 mg/g). Despite the low anthocyanins in *A. hindersonii*, the amount obtained (0.4133 mg/g) was higher than in *Myosotis sylvatica* (0.2770 mg/g) [30]. Thus, both *A. venetum* and *A. hendersonii* are good choices as an anthocyanin supplement for consumers.

## 3. Materials and Methods

### 3.1. Plant Materials and Experimental Conditions

The asexual tissue cultured *A. venetum* and *A. hendersonii* were transfer to 15 L pots and grown in an air-conditioned greenhouse under long day condition (16 h, 22 °C day; 8 h, 18 °C night). In all experiments, additional fertilizer and/or pesticides were applied to the plants when necessary. Plants with similar canopy (diameter = 1 m) were randomly selected, and the 4–6 cm long leaves which were 20 cm away from the apex on every branch from the 2-year-old plants were collected for genomic survey, LC/MS, and RNA-sequence analyses.

For LC/MS analysis, leaves from each of the eight plants of the two species were separately collected as biological replicates. All of the samples were oven-dried to a constant weight at 60 °C. The dried leaves were ground to powder using a mixer mill with zirconia bead for 1.5 min at 30 Hz, and then passed through a 1.2-mm-size mesh. One hundred milligrams of the powdered samples was separately dissolved in 1.0 mL 70% methanol at 4 °C overnight for extraction. Then, after centrifuging at 10,000× *g* for 10 min, the extracts were passed through the solid-phase extraction cartridge (ANPEL, Shanghai, China) at 3 mL/min, filtrated through a 0.22 μm pore size membrane (ANPEL, Shanghai, China), and transferred to LC vials. The vials were stored at −80 °C before LC/MS analysis. QC samples (quality control samples) were prepared by mixing aliquots of the all samples to be a pooled sample

As for genomic and transcriptome analysis, total DNA and RNA were isolated from the same plants used for LC/MS analysis. The genomic DNA and total RNA were extracted from *A. venetum* and *A. hendersonii* leaf material using the Qiagen BioSprint and Qiagen RNeasy Plant Mini Kits (Qiagen, Hilden, Germany), according to the manufacturer’s protocol, respectively, and stored at −80 °C before use. The quantity and quality of genomic DNA were measured by an ND-1000 spectrophotometer (Thermo Fisher Scientific, Waltham, CA, USA) and the RNA 6000 Nano Labchip Kit (Agilent Technologies, Santa Clara, USA) was used for total RNA measuring.

### 3.2. Genomic Survey, De Novo Transcriptome Assembly, and Analysis

Genomic paired-end libraries with 170 bp and 500 bp insertions were constructed according to the manufacturer’s standard protocol for Illumina Genome Analysis (Illumina Hiseq2000, San Diego, CA, USA). The SOAPdenovo2 software was used to carry out preliminary genome assembly [24]. The estimation of genome size, repetitive sequences, and heterozygosity were based on the information on peak depth, and the results of 25 mers Kmer analysis. The heterozygous rate estimation in the early stage of the genome project was according to the method modified and addressed by Liu et al. [31].

RNA-seq libraries were constructed using an RNA Library Prep Kit according to the manufacturer’s instruction (NEB, Ipswich, MA, USA), and the quality was measured using the Agilent Bioanalyzer 2100 system. The RNA-seq sequencing and assembly were performed by Biomarker Technology Co. (Biomarker, Beijing, China) with the Illumina HiSeq™ 2500 platform (BGI, Shenzhen, China). Prior to assembly, the raw data were filtered by trimming adaptor sequences and removing low quality sequences (Q < 20) with more than 10% uncertain (N) bases, the clean reads were then de novo assembled into unigenes according to the Trinity program [32,33].

### 3.3. Gene Function Annotation, Classification, and Digital Gene Expression Profile

Gene function was annotated according to the Nr, Nt, Pfam, Swiss-Prot, KO, and GO biological databases. For each sequence, top hits were extracted and used for further process. Differential expression analysis of two samples was performed using the DEGseq R package [34]. Based on the read count of each transcript, the reads per kilo base per million (RPKM) value was calculated and same taken to Gene Spring 12.5 GX to get fold changes (FC) through libraries with default parameter. Fold changes were calculated for category *A. venetum* vs *A. hindersonii. q*-value < 0.005 and |log2(foldchange)| ≥1 were set as thresholds for significant differential expression. Finally, sequences were filtered with RPKM ≥ 0.3, FDR > 0.001 and FC ≥ 2. The KOBAS software was used to test the statistical enrichment of the DEGs in KEGG [35].

Annotation references to flavonoid biosynthesis obtained from the database, such as gene descriptions, GO terms, and sequence descriptions, were manually classified according to the known keywords (referred by published articles and KEGG pathways) for the selection of candidate flavonoid biosynthesis-related genes. Gene expression was quantified using the Maxima SYBR Green qPCR Master Mix (Invitrogen Corp, Carlsbad, CA, USA) and the CFX-96 Thermal Cycler (Bio-Rad, Hercules, CA, USA) using primers appropriate for each gene.

### 3.4. LC/MS, Data Preprocessing, and Statistical Analysis

The ACQUITY UHPLC system (Waters Corpo MA, Milford, USA) and Triple TOF 5600 System (AB SCIEX, Framingham, MA, USA) were used to analyze metabolic profiling, and the 1.7 μm, 2.1 × 100 mm BEH C18 column was employed in both the positive and negative modes. The injection volume was 50 μL, and the binary gradient elution system consisted of water (0.1% formic acid, *v/v*) and acetonitrile (0.1% formic acid, *v/v*). The separation was achieved using the gradient (0 min, 5% acetonitrile; 2 min, 20% acetonitrile; 4 min, 25% acetonitrile; 9 min, 60% acetonitrile; 14 min, 100% acetonitrile; 18 min, 100% acetonitrile; 18.1 min, 5% acetonitrile and 19.5 min, 5% acetonitrile) with the flow rate 0.4 mL/min and column temperature 45 °C.

Data acquisition was performed in full scan mode (*m/z* ranges from 70 to 1000) combined with IDA mode. Parameters of mass spectrometry were: 550 °C (+) and 550 °C (−) ion source temperature; 5500 V (+) and 4500 V (−) ion spray voltage; 35 PSI curtain gas; 100 V (+) and −100 V (−) declustering potential; 10 eV (+) and −10 eV (−) collision energy; 550 °C (+) and 600 °C (−) interface heater temperature. The range of *m/z* was set as 25–1000, and the collision energy was set as 30 eV for IDA analysis. The QCs were injected at regular intervals (every 10 samples) throughout the analytical run to provide a set of data from which repeatability can be assessed.

The acquired LC/MS raw data were analyzed by the progqenesis QI software (Waters Corporation, Milford, MA, USA). The 0.3 mg/ml 2-Chloro-L-phenylalanine (CAS: 103616-89-3) in methanol solution was used as reference substance, and the data analysis was based on peak area normalization. The detection parameters of reference substance were deselected for peak retention time alignment with minimum intensity at 15% of base peak intensity, noise elimination level at 10.00, and isotopic peaks were excluded. The internal standard was used for data QC (RSD < 40). The metabolic alterations among different experimental groups were visualized by principle component analysis (PCA) and (orthogonal) partial least-squares-discriminant analysis (O)PLS-DA (Orthogonal-projection to latent structures discriminant analysis). After mean centering (Ctr) and Pareto variance (Par) scaling, the differential metabolites were selected on the basis of the combination of a statistically significant threshold of variable influence on projection (VIP) values. The VIP values were obtained from the OPLS-DA model and *p*-values from a two-tailed Student’s *t*-test on the normalized peak areas. The metabolites with VIP values larger than 1.0 and *p*-values less than 0.05 were considered as differential metabolites. The default seven-round cross-validation was applied with 1/seventh of the samples being excluded from the mathematical model in each round, in order to guard against overfitting.

### 3.5. Flavonoids and Total Anthocyanin Quantifiation

Quantitative analysis of flavonoids hyperoside, isoquercetrin, quercetrin, and rutin was carried out using HPLC–UV method [36]. Briefly, 0.2 g sample from *A. venetum* and *A. hendersonii* leaf was refluxed with 40 mL 70% methanol for 3 h, and then centrifuged at 3000× *g* for 5 min. After washing with 70% methanol and centrifuging at 3000× *g* for 5 min again, the extracts were marked to 50 mL volumetric flask. Subsequently, the prepared extract solution was filtered through a 0.45 μm PTFE membrane for HPLC–UV analysis. The analysis was carried out on an Agilent Zorbax column (250 × 4.6 mm, 5 μm) at 25 °C, and the Agilent 1100 liquid chromatography system equipped with a quaternary solvent delivery system.

Total anthocyanin contents (TAC) were extracted according to the pH differential method, with minor modifications [37,38,39,40]. Briefly, the main affecting factors, such as ethanol content in the solution (60–80%, *v/v*), liquid–solid ratio [40:1–60:1, *w/v* (g/mL)], leaching time (2–5 h), and temperature (30–60 °C), were taken into account. After centrifuging, the combined and filtered extraction solvent were measured at 520 and 700 nm in buffers at pH 1.0 and 4.5 using a UV spectrophotometer (DU730UV VIS, Beckman Coulter, Brea, CA, USA). Anthocyanins ((mg)/(l)) = (A × Mw × DF × 103)/(ε × L), where A is absorbance given by A = (A_520 nm–A_700 nm ) pH1.0 − (A_520 nm–A_700 nm ) pH 4.5; Mw, molecular weight of anthocyanins (433.2 g/mol); DF, dilution factor; ε, extinction coefficient (29,600 L·mol^–1^·cm^–1^); and L, path length (1 cm).

### 3.6. Transcript–Metabolite Correlation Analysis and qRT-PCR Validation of the Selected Key Genes

The entire raw transcriptome dataset and the entire metabolome dataset, formatted in log2 abundance (peak area), were uploaded to the Metabolights with repository: https://www.ebi.ac.uk/metabolights/MTBLS1393. Statistical significance of the *A. venetum* and *A. hendersonii* metabolite abundances was calculated through the PMR [41] and Metabolights [42] database.

Metabolites with *q*-values less than 0.1 were selected for transcript–metabolite correlation analysis. For each selected metabolite, their related genes were identified according to a Pearson correlation coefficient of metabolite–transcript >0.9. The identified genes or transcripts were then used to determine over-represented pathways through MetNetOnline (http://www.metnetonline.org), and the significantly enriched pathways with *p* < 0.05 were determined for every metabolite. The individual pathways and their relevant locus IDs were then ranked according to the number of metabolites associated with them [43].

Gene expression was conducted using cDNA from three biological replicates, with three technical replicates each. The expression of mRNA PAL (Gene ID: *DN7770*, *DN12095*), C4H (Gene ID: *DN15816*), CHI (Gene ID: *DN13226*, *DN3483* and *DN8427*), CHS (Gene ID: *DN18628*), F3H (Gene ID: *DN13255* and *DN9170*), ANR (Gene ID: *DN11675*, *DN11945* and *DN20760*), and UGT (Gene ID: *DN11850*, *DN14334*, *DN16440*, *DN19250* and *DN6934*) were quantified with RT-qPCR using the SYBR Green qPCR Master Mix (Invitrogen Corp) and the CFX-96 Thermal Cycler (Bio-Rad). The relative expression levels of these selected genes between *A. venetum* and *A. hendersonii* leaf were calculated using the comparative threshold cycle method [44], and the expression levels were normalized against housekeeping gene actin. The specific primers for these selected genes and actin gene are shown on Table S2.

## 4. Conclusions

In this study, the genome survey of two *Apocynum* plants, *A. venetum* and *A. hendersonii,* were conducted to provide a foundation for whole genome sequencing. Results showed both species to have small genome sizes of 232.80 Mb (*A. venetum*) and 233.74 Mb (*A. hendersonii*), suggesting future genome assembly and comparative studies on genome evolution to also be relatively simple. Transcriptome and metabolome co-analysis demonstrated that flavonols were the main differentiated flavonoids between the two species, and positive correlations of gene expression levels of flavonone-3 hydroxylase (F3H), anthocyanin reductase (ANR), flavonoid 3-O-glucosyltransferase (UGT), and total flavonoid content were observed. The content of isoquercitrin, hyperoside, and total anthocyanin in *A. venetum* was found to be much higher than in *A. hendersonii*, especially the difference of total anthocyanin content between the two species was thought to be the reason for the phenotype diversity in stem and leaf color of *A. venetum* (red) and *A. hendersonii* (white).

## Figures and Tables

**Table 1:** Statistics of high-throughput sequencing result.

Species	Raw Base (bp)	Clean Base (bp)	Clean Read Number	Clean Read Rate (%)	Raw Q30 Base Rate	Clean Q30 Base Rate
*A. venetum*	116,453,678,200	114,264,349,800	763,459,276	98.12	92.16	92.75
*A. hendersonii*	120,605,812,500	117,392,457,300	782,616,382	97.34	91.92	92.11

**Table 2:** Contig and scaffold assembled sequences in *A. venetum* and *A. hendersonii*.

Genome Characters	*A. venetum*		*A. hendersonii*	
	Contig	Scaffold	Contig	Scaffold
Total length (bp)	322,394,863	377,590,884	199,859,654	236,656,356
Total number	1250,389	876,453	741,647	303,264
Max length (bp)	6632	70,393	8521	110,436
N50 length (bp)	310	1225	457	4667
N90 length (bp)	132	150	114	217

**Table 3:** Genome characteristics of *A. venetum* and *A. hendersonii*.

Genome Characters	*A. venetum*	*A. hendersonii*
K-mer number	93,121,402,015	93,494,221,128
K-mer depth	400	400
Genome size (Mbp)	232.80	233.74
Revised genome size (Mbp)	224.36	228.21
Heterozygosity ratio (%)	0.67	0.46

**Table 4:** Flavonoids identified in the leaf extract of *A. venetum* and *A. hendersonii.*

Metabolites	Retention Time (min)	*m/z*	Formula	*A. venetum* vs. *A. hendersonii* log2 (FC, Fold Change)
1. Flavones				
Luteolin	10.377	267.031	C_15_H_10_O_6_	-
Apigenin	3.338	271.060	C_15_H_10_O_5_	-
2. Flavonols				
Rutin	3.430	591.138	C_27_H_30_O_16_	1.788
Hyperin	3.313	465.103	C_21_H_20_O_12_	3.452
Isoquercitrin	3.314	463.087	C_21_H_20_O_12_	2.955
Quercetin	3.460	303.050	C_15_H_10_O_7_	7.967
Kaempferol	4.066	285.039	C_15_H_10_O_6_	4.030
Tamarixetin	3.550	363.07	C_16_H_14_O_7_	-
3. Flavanones				
Hesperidin	3.632	591.172	C_28_H_34_O_15_	−2.302
4. Isoflavanone				
Trifolin	3.479	429.083	C_21_H_20_O_11_	-
5. Flavanols				
Epigallocatechin	2.463	307.081	C_15_H_14_O_7_	−0.144
Catechin	2.977	883.210	C_45_H_38_O_19_	−1.001
Epicatechin	3.763	271.062	C_15_H_14_O_6_	-
6. Anthocyanidins				
Cyanidin	3.216	287.055	C_43_H_42_O_22_	1.952
Procyanidin C1	3.097	867.215	C_15_H_10_O_6_	0.959
Procyanidin	3.017	579.150	C_45_H_38_O_18_	-
Delphinidin	3.313	303.050	C_15_H_10_O_7_	0.118
Pelargonidin	11.853	611.049	C_15_H_11_ClO_5_	-
Malvidin	11.268	347.033	C_17_H_15_ClO_7_	-
Peonidin	10.826	381.038	C_16_H_13_ClO_6_	-
7. Others				
Eriodictyol	3.001	577.135	C_15_H_12_O_6_	−1.042
Acacetin-7-O-rutinoside	4.261	677.247	C_25_H_24_O_14_	2.051
Chrysoeriol 7-O-glucoside	3.583	547.109	C_25_H_24_O_14_	4.863
Carthamin	2.463	909.210	C_43_H_42_O_22_	-
Neocarthamin	3.518	449.108	C_21_H_22_O_11_	−0.740

**Table 5:** Quantitative analysis result of flavonoids and total anthocyanins in *A. venetum* and *A. hendersonii* leaves. Values are reported as mean ± standard (SD) of two independent experiments performed in triplicate.

Species	Hypersoside (mg/g Dry Weight)	Isoquercitrin (mg/g Dry Weight)	Quercetin (mg/g Dry Weight)	Rutin (mg/g Dry Weight)	Total Anthocyanins (mg/g Dry Weight)
*A. venetum*	3.896 ± 0.043	5.254 ± 0.057	0.543 ± 0.036	3.653 ± 0.012	0.9017 ± 0.002
*A. hendersonii*	1.262 ± 0.025	1.718 ± 0.029	0.384 ± 0.015	2.275 ± 0.015	0.4133 ± 0.002

## Supplementary Materials

The following are available online at https://www.mdpi.com/2218-1989/9/12/296/s1, Figure S1: Multivariate statistical analysis. PCA score plots (A-B); PLS-DA (C); OPLS-DA (D-E), Table S1: Confirmation the expression level of selected key genes from *A. venetum* vs *A. hendersonii* transcriptomes by quantitative PCR, Table S2: Appropriate qRT-PCR primers for selected genes and house keeping gene actin.

## References

[1] Grundmann O., Nakajima J.I., Kamata K., Seo S., Butterweck V. (2009). Kaempferol from the leaves of Apocynum venetum possesses anxiolytic activities in the elevated plus maze test in mice. Phytomedicine.

[2] Lu C., Zhang W., Peng X., Gu G., Chen M., Tang Z. (2010). Development of Randomly Amplified Polymorphic DNA-Sequence Characterized Amplified Region Marker for Identification of Apocynum venetum LINN. from A. pictum SCHRENK. Biol. Pharm. Bull..

[3] Thevs N., Zerbe S., Kyosev Y., Rozi A., Tang B., Abdusalih N., Novitskij Z. (2012). Apocynum venetum L. and Apocynum pictum Schrenk (Apocynaceae) as multi-functional and multi-service plant species in Central Asia: A review on biology, ecology, andutilization. J. Appl. Bot. Food Qual..

[4] Kasimu R., Fan Z., Wang X., Hu J., Wang P., Wang J. (2015). Anti-platelet aggregation activities of different fractions in leaves of *Apocynum venetum* L.. J. Ethnopharmacol..

[5] Wu T., Li X., Li T., Cai M., Yu Z., Zhang J., Zhang Z., Zhang W., Xiang J., Cai D. (2018). Apocynum venetum Leaf Extract Exerts Antidepressant-Like Effects and Inhibits Hippocampal and Cortical Apoptosis of Rats Exposed to Chronic Unpredictable Mild Stress. Evid. Based Complement. Altern. Med..

[6] Lau Y.S., Kwan C.Y., Ku T.C., Hsieh W.T., Wang H.D., Nishibe S., Dharmani M., Mustafa M.R. (2012). Apocynum venetum leaf extract, an antihypertensive herb, inhibits rat aortic contraction induced by angiotensin II: A nitric oxide and superoxide connection. J. Ethnopharmacol..

[7] Chan C.O., Lau C.C., Ng Y.F., Xu L., Chen S.B., Chan S.W., Mok K.W. (2015). Discrimination between Leave of Apocynum venetum and Its Adulterant, A. pictum Based on Antioxidant Assay and Chemical Profiles Combined with Multivariate Statistical Analysis. Antioxidants.

[8] Xie W., Zhang X.Y., Wang T., Hu J. (2012). Botany, traditional uses, phytochemistry and pharmacology of Apocynum venetum L. (Luobuma): A review. J. Ethnopharmacol..

[9] Yokozawa T., Kashiwada Y., Hattori M., Chung H.Y. (2012). Study on the components of luobuma with peroxynitrite-scavenging activity. Biol. Pharm. Bull..

[10] Zhang W.O., Peng X.M., Lu C.M., Wang M.L., Gu G.P. (2007). A taxonomic study of luonuma based on sequence data of 3 non-coding DNA regions. Acta Bot. Boreali-Occident. Sin..

[11] Bogs J., Ebadi A., McDavid D., Robinson S.P. (2006). Identification of the flavonoid hydroxylases from grapevine and their regulation during fruit development. Plant Physiol..

[12] Boss P.K., Davies C., Robinson S.P. (1996). Analysis of the expression of anthocyanin pathway genes in developing Vitis vinifera L. cv shiraz grape berries and the implications for pathway regulation. Plant Physiol..

[13] Castellarin S.D., Pfeiffer A., Sivilotti P., Degan M., Peterlunger E., Gaspero G.D. (2010). Transcriptional regulation of anthocyanin biosynthesis in ripening fruits of grapevine under seasonal water deficit. Plant Cell Environ..

[14] Garcia S., Leitch I.J., Anadon-Rosell A., Canela M., Gálvez F., Garnatje T., Gras A., Hidalgo O., Bennett M.D. (2014). Recent updates and developments to plant genome size databases. Nucleic Acids Res..

[15] Leitch I.J., Leitch A.R. (2013). Genome Size Diversity and Evolution in Land Plants. Plant Genome Divers..

[16] Jiao W.B., Huang D., Xing F., Hu Y., Deng X.X., Xu Q., Chen L.-L. (2013). Genome-wide characterization and expression analysis of genetic variants in sweet orange. Plant J..

[17] Yang J., Zhao X., Ke C., Du H., Ouyang Y., Chen J., Qiu S., Huang J., Jiang Y., Jiang L. (2012). A killer-protector system regulates both hybrid sterility and segregation distortion in rice. Science.

[18] Ogata H., Goto S., Sato K., Fujibuchi W., Bono H., Kanehisa M. (1999). KEGG: Kyoto encyclopedia of genes and genomes. Nucleic Acids Res..

[19] Belmonte V.N., Retamal M., Mezcua M., Fernández-Alba A.R. (2012). A sensitive and selective method for the determination of selected pesticides in fruit by gas chromatography/mass spectrometry with negative chemical ionization. J. Chromatogr. A.

[20] Liang S., Zhao L., Lv L., Zhang H., Guo T., Chai Y., Zhang G. (2014). Screening and analysis of metabolites in rat urine after oral administration of Apocynum venetum L. extracts using HPLC–TOF-MS. J. Sep. Sci..

[21] Yan S.X., Lang J.L., Song Y.Y., Wu Y.Z., Lv M.H., Zhao X., Liu Y.H., Xu C.Y. (2015). Studies on Anti-Depressant Activity of Four Flavonoids Isolated from Apocynum venetum Linn (Apocynaceae) Leaf in Mice. Trop. J. Pharm. Res..

[22] Schaefer H., Schaefer V., Levey D. (2004). How plant-animal interactions signal new insights in communication. Trends Ecol. Evol..

[23] Bendokas V., Skemiene K., Trumbeckaite S., Stanys V., Passamonti S., Borutaite V., Liobikas J. (2019). Anthocyanins: From plant pigments to health benefits at mitochondrial level. Crit. Rev. Food Sci. Nutr..

[24] Pertea G., Huang X., Liang F., Antonescu V., Sultana R., Karamycheva S., Lee Y., White J., Cheung F., Parvizi B. (2003). TIGR Gene Indices clustering tools (TGICL): A software system for fast clustering of large EST datasets. Bioinformatics.

[25] Espley R.V., Hellens R.P., Putterill J., Stevenson D.E., Kutty-Amma S., Allan A.C. (2010). Red colouration in apple fruit is due to the activity of the MYB transcription factor, MdMYB10. Plant J..

[26] Wang Y., Xu H., Wang N., Jiang S., Liu J., Wang D., Zuo W., Chen X. (2017). Molecular cloning and expression analysis of an auxin signaling related gene mdarf3 in red flesh apple. Acta Hortic. Sin..

[27] Wang N., Jiang S., Zhang Z., Fang H., Xu H., Wang Y., Chen X. (2018). Malus sieversii: The origin, flavonoid synthesis mechanism, and breeding of red-skinned and red-fleshed apples. Hortic. Res..

[28] Su M., Wang N., Jiang S., Fang H., Xu H., Wang Y., Zhang Z., Zhang J., Xu L., Zhang Z. (2018). Molecular characterization and expression analysis of the critical floral gene mdagl24-like in red-fleshed apple. Plant Sci..

[29] Dong T., Han R., Yu M., Zhang Y., Gong Y., Li Z. (2018). Anthocyanins accumulation and molecular analysis of correlated genes by metabolome and transcriptome in green and purple asparaguses (*Asparagus officinalis*, L.). Food Chem..

[30] Huang X., Zou X., Zhao J., Shi J., Zhang X., Mel H. (2014). Measurement of total anthocyanins content in flowering tea using near infrared spectroscopy combined with ant colony optimization models. Food Chem..

[31] Ye J., Zhang Y., Cui H., Liu J., Wu Y., Cheng Y., Xu H., Huang X., Li S., Zhou A. (2018). Wego 2.0: A web tool for analyzing and plotting go annotations, **2018** update. Nucleic Acids Res..

[32] Liu B., Shi Y., Yuan J., Hu X., Zhang H., Li N., Li Z., Chen Y., Mu D., Fan W. (2013). Estimation of genomic characteristics by analyzing k-mer frequency in de novo genome projects. arXiv.

[33] Grabherr M.G., Haas B.J., Yassour M., Levin J.Z., Thompson D.A., Amit I., Adiconis X., Fan L., Raychowdhury R., Zeng Q. (2011). Full-length transcriptome assembly from RNA-Seq data without a reference genome. Nat. Biotechnol..

[34] Wang L., Feng Z., Wang X., Wang X., Zhang X. (2010). DEGseq: An R package for identifying differentially expressed genes from RNA-seq data. Bioinformatics.

[35] Xie C., Mao X., Huang J., Ding Y., Wu J., Dong S., Kong L., Gao G., Li C., Wei L. (2011). KOBAS 2.0: A web server for annotation and identification of enriched pathways and diseases. Nucleic Acids Res..

[36] Sagaradze V.A., Babaeva E.Y., Kalenikova E.I. (2017). HPLC-UV method for determing flavonoids in hawthorn flowers and leaves. Pharm. Chem. J..

[37] Patil G., Madhusudhan M.C., Babu B.R., Raghavarao K. (2009). Extraction, dealcoholization and concentration of anthocyanin from red radish. Chem. Eng. Process. Process Intensif..

[38] Tsantili E., Shin Y., Nock J.F., Watkins C.B. (2010). Antioxidant concentrations during chilling injury development in peaches. Postharvest Biol. Technol..

[39] White B.L., Howard L.R., Prior R.L. (2011). Impact of Different Stages of Juice Processing on the Anthocyanin, Flavonol, and Procyanidin Contents of Cranberries. J. Agric. Food Chem..

[40] Inácio M.R., De-Lima K.M., Michchell G., Lopes V.G., Pessoa J.D., Henrique D.A. (2013). Total anthocyanin content determination in intact a ç aí (Euterpe oleracea Mart.) and palmitero-juara (Euterpe edulis Mart.) fruit using near infrared spectroscopy (NIR) and multivariate calibration. Food Chem..

[41] Hur M., Campbell A.A., Almeida-de-Macedo M., Li L., Ransom N., Jose A., Crispin M., Nikolau B.J., Wurtele E.S. (2013). A global approachto analysis and interpretation of metabolic data for plant natural product discovery. Nat. Prod. Rep..

[42] Haug K., Salek R.M., Conesa P., Hastings J., de Matos P., Rijnbeek M., Mahendraker T., Williams M., Neumann S., Rocca-Serra P. (2013). MetaboLights—An open-access general-purpose repository for metabolomics studies and associated meta-data. Nucleic Acids Res..

[43] Reem N.T., Chen H.Y., Hur M., Zhao X., Wurtele E.S., Li X., Li L., Zabotina O. (2018). Comprehensive transcriptome analyses correlated with untargeted metabolome reveal differentially expressed pathways in response to cell wall alterations. Plant Mol. Biol..

[44] Schmittgen T.D., Livak K.J. (2008). Analyzing Real-Time PCR Data by the Comparative C(T) Method. Nat. Protoc..

