# Supplementary material for: Genomic Survey, Transcriptome, and Metabolome Analysis of Apocynum venetum and Apocynum hendersonii to Reveal Major Flavonoid Biosynthesis Pathways
Source: Metabolites. 2019 Dec 5;9(12):296. doi: 10.3390/metabo9120296 (PMC6950674; doi:10.3390/metabo9120296)
Supplement: Supplementary file 1 [file metabolites-09-00296-s001.pdf]

Table S1. Confirmation the expression level of selected key genes from *A. venetum* vs *A. hendersonii* transcriptomes by quantitative PCR.

Gene name	Gene ID	Fold change	Log2 fold Change	Up-down
flavonol 3-hydroxylase	DN13255	3.381	1.757	up
	DN9170	5.154	2.365	up
	DN11657	3.136	1.6489	up
anthocyanidin reductase	DN11954	7.130	2.833	up
	DN20760	3.493	1.804	up
	DN11850	25.055	4.647	up
	DN14334	6.473	2.694	up
flavonoid 3-O-glucosyltransferase	DN16440	2.785	1.477	up
	DN16934	5.215	2.382	up
kaempferol 3-O-beta-D-galactosyltransferase	DN10026	33.954	5.085	up
Leucoanthocyanidin reductase	DN11675	3.136	1.648	up
	DN7770	1.001	0.001	-
phenylalanine ammonialyase	DN12095	1.421	0.507	-
	DN14363	0.904	-0.144	-
cinnamic acid 4-hydroxylase	DN15816	0.831	-0.268	-
	DN13226	1.0201	0.265	-
chalcone isomerase	DN3483	1.306	0.0385	-
	DN8427	0.769	-0.378	-
chalcone synthase	DN18628	0.884	-0.176	-

Table S2. Appropriate qRT-PCR primers for selected genes and house keeping gene actin.

Models									
No.	Model	Type	A	N	R2X(cum)	R2Y(cum)	Q2(cum)	R2	Q2
ALL	M1	PCA-X	3	21	0.555	-	0.324	-	-
	M2	PCA-X	2	16	0.507	-	0.307	-	-
A.venetum/ A.hendersonii	M3	PLS-DA	3	16	0.765	0.995	0.979	-	-
	M4	OPLS-DA	1+2+0	16	0.765	0.995	0.976	0.764	0.525

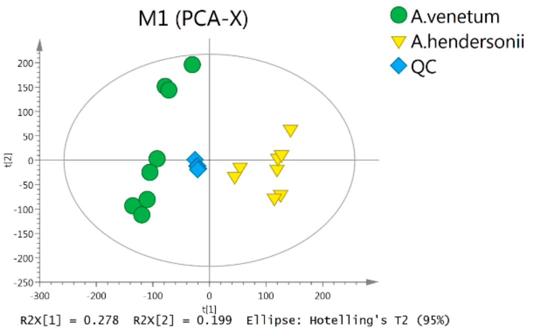

A

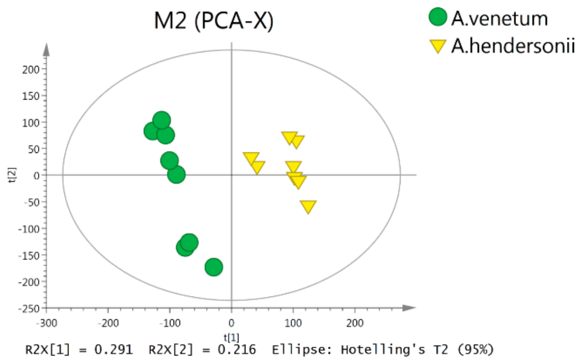

B

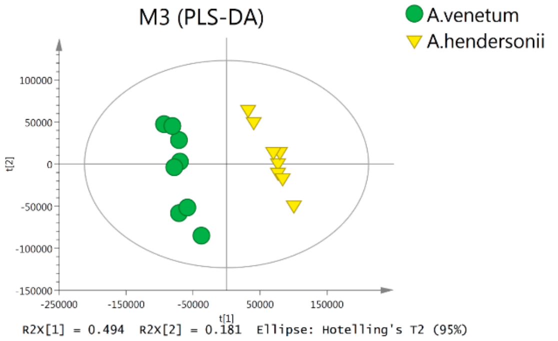

C

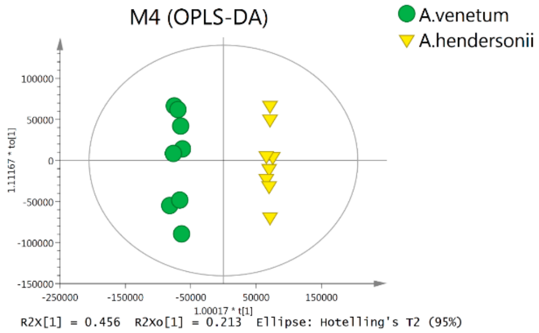

D

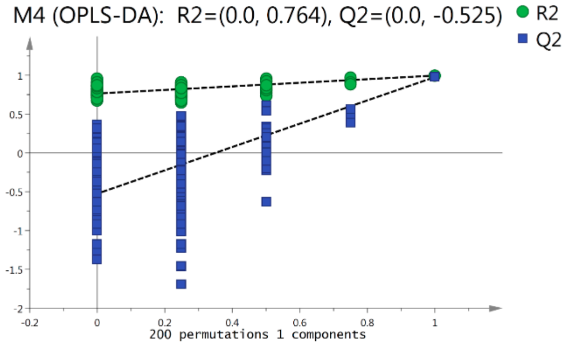

E

Figure S1. Multivariate statistical analysis. PCA score plots (A-B); PLS-DA (C); OPLS-DA (D-E);.
